# Improved responsiveness and reduced sample size requirements of PROMIS physical function scales with item response theory

**DOI:** 10.1186/ar3461

**Published:** 2011-09-14

**Authors:** James F Fries, Eswar Krishnan, Matthias Rose, Bharathi Lingala, Bonnie Bruce

**Affiliations:** 1 Department of Medicine, Stanford University School of Medicine, 1000 Welch Road, Suite 203, Palo Alto, CA 04304, USA; 2 University of Massachusetts Boston, 100 Morrissey Blvd., Boston, MA 02125-3393, USA

## Abstract

**Introduction:**

The Health Assessment Questionnaire Disability Index (HAQ) and the SF-36 PF-10, among other instruments, yield sensitive and valid Disability (Physical Function) endpoints. Modern techniques, such as Item Response Theory (IRT), now enable development of more precise instruments using improved items. The NIH Patient Reported Outcomes Measurement Information System (PROMIS) is charged with developing improved IRT-based tools. We compared the ability to detect change in physical function using original (Legacy) instruments with Item-Improved and PROMIS IRT-based instruments.

**Methods:**

We studied two Legacy (original) Physical Function/Disability instruments (HAQ, PF-10), their item-improved derivatives (Item-Improved HAQ and PF-10), and the IRT-based PROMIS Physical Function 10- (PROMIS PF 10) and 20-item (PROMIS PF 20) instruments. We compared sensitivity to detect 12-month changes in physical function in 451 rheumatoid arthritis (RA) patients and assessed relative responsiveness using *P*-values, effect sizes (ES), and sample size requirements.

**Results:**

The study sample was 81% female, 87% Caucasian, 65 years of age, had 14 years of education, and had moderate baseline disability. All instruments were sensitive to detecting change (< 0.05) in physical function over one year. The most responsive instruments in these patients were the Item-Improved HAQ and the PROMIS PF 20. IRT-improved instruments could detect a 1.2% difference with 80% power, while reference instruments could detect only a 2.3% difference (*P *< 0.01). The best IRT-based instruments required only one-quarter of the sample sizes of the Legacy (PF-10) comparator (95 versus 427). The HAQ outperformed the PF-10 in more impaired populations; the reverse was true in more normal populations. Considering especially the range of severity measured, the PROMIS PF 20 appears the most responsive instrument.

**Conclusions:**

Physical Function scales using item improved or IRT-based items can result in greater responsiveness and precision across a broader range of physical function. This can reduce sample size requirements and thus study costs.

## Introduction

Successful management of chronic illnesses, such as rheumatoid arthritis (RA), depends in part upon instruments that validly and precisely measure Physical Function (PF) and can guide appropriate intervention. In RA, Physical Function is commonly assessed by the Health Assessment Questionnaire Disability Index (HAQ or HAQ-DI) [1] and the SF-36 PF-10 [2], both developed three decades ago using classical test theory. Consequently, they were not subjected to modern psychometric approaches, including study of domain definitions, item context and difficulty, time frame, response options, clarity, importance, or information content. Modern methods, like Item Response Theory (IRT), quantitatively assess item properties and identify items with the highest information content [3,4], enabling development of more precise instruments [4,5].

Improved instrument sensitivity can result from using IRT-based item calibrations, selection of the best items for new instruments, and using individually tailored items for specific uses. Better precision can increase statistical power or hold statistical power constant while decreasing questionnaire burden [6,7].

The Patient Reported Outcome Measurement Information System (PROMIS) is an NIH Roadmap infrastructure project charged with developing IRT-based patient reported outcome instruments for improved validity and efficiency in experimental and observational studies [8,9]. Physical Function is one of the initial PROMIS health domains and is defined as "the ability to perform activities of daily living (ADL) and instrumental activities of daily living" (IADL) [10]. It is analogous to most existing instruments, including the original (Legacy) HAQ [1,11] and PF-10 [2]. The term "Physical Function" is favored by PROMIS over "Disability" [12], since "Disability" refers only to decrements of function below the population mean while "Physical Function" includes abilities both above and below the mean. The PROMIS conceptual framework includes Physical Function from functional loss to activity and participation issues and is consistent with the WHO International Classification of Function (ICF) model [13].

The 154-item PROMIS PF item bank was developed using classic and modern item assessment methods [12,14] and includes the 20-item stems of the original HAQ and the 10-item stems of the original PF10. Physical Function items (*n *= 1,860) were aggregated from exhaustive literature searches and evaluated for attributes including clarity, importance and comprehension, uni-dimensionality, independence, item difficulty, and item information content and were calibrated on more than 20,000 normal and diseased participants [4,12].

This study was designed to determine comparative responsiveness and effects on sample size requirements between Legacy, Item-Improved and PROMIS IRT-based instruments. We hypothesized that the modified new instruments would be sensitive to change over 12 months, would assess responsiveness (sensitivity to change) as well or better than Legacy instruments, would be applicable to greater ranges of disease severity, and would require smaller sample sizes than Legacy instruments.

## Materials and methods

### Participants

English-speaking participants met American College of Rheumatology criteria for RA. Of 521 participants completing initial questionnaires, we studied the 451 (87%) who returned both baseline and 12-month questionnaires. Respondents were 81% female, 87% Caucasian, averaged 65 years of age with 14 years of education, and had a baseline Legacy HAQ score averaging 0.88. The Stanford Institutional Review Board approved the study, and all subjects gave written informed consent.

### Instruments

We studied six instruments: the (1) Legacy HAQ and (2) PF-10, (3) Item-Improved HAQ and (4) Item-Improved PF-10, and the (5) PROMIS PF-10 and (6) PROMIS PF-20. The Legacy HAQ and PF-10 ask about ability over the past week. The Legacy HAQ contains 20 items in eight categories (dressing and grooming, arising, eating, walking, hygiene, reach, grip and activities) with four response options: "without any difficulty," "with some difficulty," "with much difficulty," "unable to do." The Legacy PF-10 contains 10 items with three response options: "yes, limited a lot," "yes, limited a little," and "no, not limited at all".

The IRT-based instruments, the Item-Improved 20-item HAQ and 10-item PF-10 and the PROMIS PF-10 and PF-20 all assess present abilities (for example, Are you able to walk a block?). Both "Item-Improved instruments" have five-response options: HAQ - "without any difficulty," "with a little difficulty," "with some difficulty," "with much difficulty," "unable to do"; PF-10 - "not at all," "very little," "somewhat," "quite a lot," "cannot do." We term these items "improved" since PROMIS studies document that present tense and a five-item response set improve instrument responsiveness. Thus, the "item-improved" instruments retain the stem of the original items, but have "improved" the tense to "present" and the response categories to "five" (together with minor improvements in clarity and phrasing). The PROMIS PF 10 and 20 contain IRT-based items selected from the PROMIS PF 154 item bank and include Item-Improved HAQ and PF-10 items. The "IRT-based" PROMIS PF 10 and 20 are selected using IRT information content data from over 21,000 subjects to identify the best items and minor qualitative input to ensure that all major areas of Physical Function/Disability were represented in the instrument, with their strongest items. The PF 10 is a subset of the best items in the PF 20.

For the analyses which follow, we scored all instruments as the average of all items, either 10 or 20. All instruments were scored on a 0 to 100 scale (100 = worst disability) averaging scores of component items, and reversing direction as appropriate. Participants also completed validated pain and global health scales [15,16]. The original Legacy instruments, HAQ and PF-10, were administered and scored, then scores were arithmetically adjusted to the 0 to 100 scale. Item-Improved instruments were changed as appropriate under PROMIS protocols for clarity, translatability with focus groups, cognitive reviews, and cognitive surveys including the patient perspective [10]. The item content was unchanged from the Legacy instruments but the time frame was moved to the present tense from "over the past week" and the response options changed from four to five by insertion of an option "with a little difficulty" intended to increase sensitivity and to reduce ceiling effects. The PROMIS 10 and 20 item instruments included both Item-Improved old and new items from the 154 item bank. All items contained the five response options and present tense. The PROMIS 10-item set is contained within the PROMIS 20-item set.

The PROMIS PF-10 instrument contains five original HAQ derivatives and five original PF-10 derivatives selected from the 30 items using IRT and content balancing to include the better functioning items. The PROMIS PF 20 instrument contains six items derived from the HAQ, five from the PF-10, and nine new items from the 124 items in the PROMIS physical function item bank.

Items were selected for IRT information content after consideration of content balancing issues by a study group led by M. Rose. These methods were expected to improve all of the original items to a greater or lesser extent, and to improve the modified scales whenever a new item with higher IRT scores replaced a Legacy item.

Changes thus are considered evolutionary and to remain buttressed by the hundreds of validation studies that have been performed on the Legacy instruments [1,2,10-12]. The primary objective was to show sensitivity of the new instruments to be equivalent (non-inferior) to benchmark instruments. The secondary objective was to explore areas where the new scales might actually be superior to the old (for example, normal or severely impaired populations). Because of the small effect sizes expected when comparing instruments rather than interventions, statistical power may be marginal at best. Computerized Adaptive Testing (CAT) applications under evaluation currently utilize the entire 154-item pool and should result in substantial further improvement [8].

### Implementation

We assessed participants using mailed questionnaires, randomly ordering instruments within questionnaires to eliminate order effects. We followed established Arthritis Rheumatism and Aging Medical Information System protocols for follow up and quality control [17].

### Endpoints

The study endpoints were the 12-month change in scores using each instrument, representing an average progression of disability in rheumatoid arthritis over a year. We studied the differences between instruments rather than treatments; thus the effect-size is expected to be much smaller than the effect of an RA treatment. Observed change scores are a function of the "true change" and of the error terms surrounding estimation of the baseline and follow-up values. A more precise instrument will have smaller standard errors, allowing a more precise estimation of the true change. We used the quantitative one-year change in Physical Function scores under usual care; these changes include the progression of RA, the progression of co-morbid chronic illness, and the progression of frailty related to aging.

### Metrics

#### Responsiveness

We assessed participants' 12-month Physical Function change score from baseline and between instruments (relative instrument sensitivity) with exact *P*-values (pair-wise t-tests), computed Cohen's [18] and Guyatt's [19] effect sizes (ES) (mean change from baseline divided by baseline standard deviation (SD) or change score SD), the standardized response mean (SRM) (mean change score divided by SD of the change) [19], and the minimum detectable difference (MDD) [20].

#### Sample size

For between-instrument comparisons, we computed sample sizes required per study arm for 80% power, with an alpha of 0.05, given the delta that was observed. We used bootstrapping methods to determine confidence limits for the change score SDs and computed the confidence limits for sample size requirements from the confidence limits of the change score SD.

#### Statistics

Data are described by proportions and means (± SD). We defined non-inferiority as when neither instrument was statistically superior to the other at the *P *< 0.05 level given 80% study power. Power for this study is more than sufficient to detect a change equal to or greater than the minimal clinically important difference (MCID) for Physical Function, consistently cited for the HAQ at 0.22 units or 7% of the possible change [21]. We used the same 7% of possible change estimate for each instrument since all were estimating the same latent trait in the same subjects.

Since all instruments were scored identically and resulted in similar change scores, the exact level of MCID chosen is of only peripheral relevance. The minimum detectible difference (MDD) is more directly relevant to correlations between change scores of the six instruments.

## Results

Correlations between instruments were strong and all changes were in the same direction and of similar magnitude, indicating that all instruments are estimating the same, or a similar, latent trait. Differences between instruments are consistent with the SD of the change score, which is smaller in the Item-Improved and IRT-based scales.

Table 1 presents baseline and 12-month scores, change scores, and significance levels. Pain (*P *= 0.31) and global health scores (*P *= 0.02) were additionally studied as reference domains. All instruments were highly responsive to detection of change in function over 12 months (*P *< 0.05), except for the pain scale. With the Legacy and Item-Improved HAQ instruments, there was little difference in scoring by averaging eight categories versus 20 items, and we present the average scores for all items for all instruments studied to facilitate comparisons, as recommended by PROMIS. [10] Standard deviations of the mean difference are substantially reduced with some instruments as compared with others. These differences are reflected in *P*-values but more importantly in the metrics which follow.

**Table 1:** Physical function instruments, baseline, 12-month, and change scores

					Change Scores (12 months - Baseline)		
	Items (n)	Response options (n)	Baseline mean ± SD^1^	12-Months Mean ± SD	Mean	SD (95% CL^2 ^for SD)	***P*-value**^3^
**Legacy PF-10**	10	3	47.2 ± 29	49.0 ± 29	1.8	18.4 (16.5, 20.2)	0.04
**Legacy HAQ**	20	4	29.2 ± 23	30.7 ± 24	1.5	10.6 (9.4, 11.8)	0.002
**Item-Improved PF-10**	10	5	41.5 ± 26	43.0 ± 27	1.5	16.3 (13.9, 18.6)	0.05
**Item-Improved HAQ**	20	5	21.0 ± 21	22.1 ± 21	1.1	8.6 (7.7, 9.6)	0.01
**PROMIS PF 10**	10	5	33.9 ± 22	35.2 ± 23	1.3	11.1 (10.0, 12.2)	0.01
**PROMIS PF 20**	20	5	30.6 ± 21	31.8 ± 22	1.2	9.4 (8.4, 10.1)	0.01

^1 ^Standard Deviation, ^2 ^Confidence Limits, ^3 ^Pair-wise t-test.

HAQ, health assessment questionnaire; Item-Improved, Changes to items intended to improve their performance by altering parameters such as time frame and response options while retaining the same item stem; PF, physical function; PROMIS, Patient-Reported Outcomes Measurement Information System.

Table 2 displays the SRM, ES, MDD, and sample size requirements per study arm that are sufficient to detect a change score of 2.5 units on a 0 to 100 scale. Although the score progression ES over 12 months in RA is small (0.05 to 0.06), all instruments were readily able to detect this difference. Sample size requirements vary widely, ranging from 95 to 427. The largest sample sizes are for the Legacy and Improved PF-10. The Item-Improved PF-10 reduced sample sizes by approximately 90, about one fourth, compared with its parent. The Item-Improved HAQ reduces sample size by one-third, compared with its parent. The PROMIS PF 20 and the Item-Improved HAQ have similar sample size requirements in this population. Twenty-item scales outperform 10 item scales, item-improved scales outperform their parent Legacy Forms, and five response options outperform four.

**Table 2:** Physical function instruments, metric scores, and sample size requirements

	Standardized response mean	Cohen's effect size	Guyatt's effect size	Minimum detectable difference	Sample size requirement n (95% CL^1^)
Legacy PF-10	0.10	0.06	0.49	2.43	427 (345, 514)
Legacy HAQ	0.14	0.06	0.52	1.40	143 (113, 176)
Item-Improved PF-10	0.09	0.05	0.59	2.16	336 (245, 436)
Item-Improved HAQ	0.13	0.05	0.73	1.14	95 (76, 117)
PROMIS PF 10	0.13	0.05	0.52	1.47	157 (127, 189)
PROMIS PF 20	0.13	0.05	0.63	1.24	113 (90, 141)

**^1 ^**Confidence Limits

HAQ, health assessment questionnaire; Item-Improved, Changes to items intended to improve their performance by altering parameters such as time frame and response options while retaining the same item stem; PF, physical function; PROMIS, Patient-Reported Outcomes Measurement Information System.

**Figure 1:**
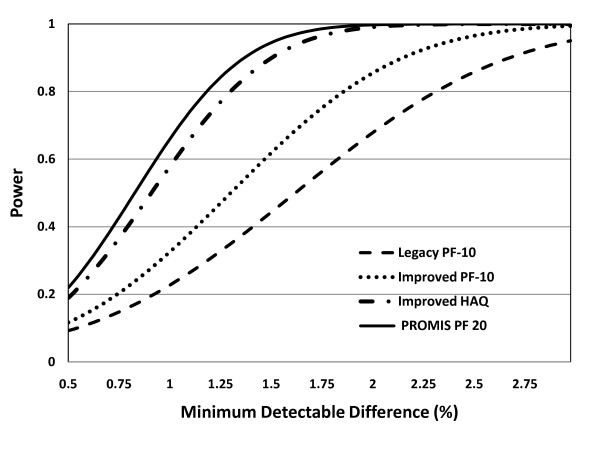
**Power versus minimum detectable difference (MDD)**. Study power as a function of the MDD, computed as the percent difference for four instruments in 451 RA patients. The Item-Improved HAQ and PROMIS PF 20 show greater power across most MDD levels. The Item-Improved PF-10 is substantially more informative than the Legacy PF-10. The maximal power difference between the greater and lesser curves is with an MDD of 1 to 1.5 percent; instruments behave more similarly when the MDD is larger or smaller. Instruments that are more sensitive have their greatest advantages in detecting small differences; when differences are large, there is less difference between individual instruments. HAQ, health assessment questionnaire; PF, physical function; PROMIS, Patient-Reported Outcomes Measurement Information System; RA, rheumatoid arthritis.

Figure 1 shows study power as a function of the MDD for four of the instruments. The more sensitive instruments are best at detecting smaller differences. When differences are large enough, there is little difference between instruments.

## Discussion

The fit between the severity distribution of the study population and the range of coverage of the instrument is centrally relevant to responsiveness, standard deviation of change, and sample size requirement issues. An instrument with items about eating, dressing, and grooming will not be sensitive to change in a population of college athletes since most will be able to do these activities easily. An instrument with items based around strenuous and difficult items about walking and climbing stairs will not perform well in a population with longstanding RA since most will have difficulty in many categories.

**Figure 2:**
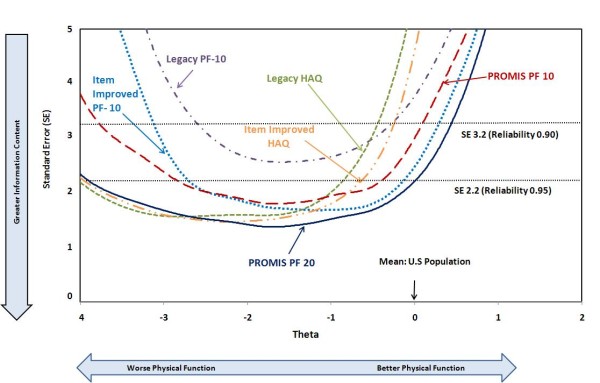
**Instrument sensitivity and disease severity**. Physical Function, on the horizontal scale, is mapped against sensitivity (reliability) on the vertical scale. A better scale has a greater breadth of Physical Function ability. The lower the curve, the greater the sensitivity. Across the overall range, the PROMIS PF 20 is most sensitive, maintaining a reliability of 0.95 over more than four SD. The Item-Improved HAQ is superior to the Item-Improved PF-10 in populations with function more than 1.2 SD from the mean (for example, Rheumatoid Arthritis) whereas the opposite is true in populations with functional abilities better than this (for example, general population-based). HAQ: health assessment questionnaire; Item-Improved: Changes to items intended to improve their performance by altering parameters such as time frame and response options while retaining the same item stem; PF, physical function; PROMIS: Patient-Reported Outcomes Measurement Information System; SD, standard deviation.

To illustrate the role of floor and ceiling effects in reducing study power, Figure 2 shows data for over 18,000 PROMIS participants from these six instruments, drawn from an approximately normal population [10]; these are sometimes termed "boat diagrams". The horizontal axis represents different functional abilities with zero representing the population mean, and each unit to the left representing one SD below the mean. Each unit to the right represents one SD above the mean. The vertical axis represents the standard error (instrument reliability), a sensitivity criterion, shown with reference reliabilities of 0.90 and 0.95. Instruments yield information curves that are more informative at some physical impairment (theta) levels than others. Above the population mean, all current instrument curves rapidly lose their sensitivity and rise steeply, representing a ceiling effect. Below the mean, some curves lose power at about three SDs, while others maintain sensitivity to beyond four SDs. Depending on the severity distribution in a sample sensitivity for a given instrument will be higher or lower. Item-Improved and IRT-based observed instrument improvement is largely due to better coverage of areas of lesser impairment.

The Item-Improved HAQ outperforms the Item-Improved PF-10 when functional abilities are more than 1.4 SD units below the population mean, while the opposite is true when abilities are better (Figure 2). The PROMIS PF 20 combines these attributes and has the greatest sensitivity across the widest range of Physical Function.

Item-improvement processes and use of IRT-based items can lead to improved instrument performance. In turn, this can make clinical research more efficient and less costly by reducing required sample sizes. The Legacy PF-10 is the most extensively used Physical Function scale and is a valid benchmark for assessing change. However, it is limited, having only 10 items and three response options, which render it less sensitive than 20-item scales or scales with five response options.

The Item-Improved HAQ performed essentially as well as the PROMIS PF 20 in the RA patients studied. This initially unexpected result was partly due to identical response options in the two instruments and to a number of shared items. More importantly, however, these RA patients had average disease severity one to two SDs below the mean, where the HAQ is most sensitive. Where the PROMIS PF 20 is strongest in comparison to the HAQ is in the one-half SD range near the population mean (Figure 2), a population not included here. The PROMIS PF 20, therefore, may be expected to outperform the HAQ in such populations [5,8,9].

The Item-Improved PF-10 outperformed the Item-Improved HAQ in normal populations. Of historical interest, the Legacy HAQ (perhaps not accidentally) has generally been utilized for RA and other serious chronic illness, and the PF-10 for more normal populations. The IRT-based PROMIS PF 20, considering all levels of impairment, outperforms the other instruments.

PROMIS and other CAT applications, better at estimating function at the extremes, should provide substantial further improvement [8,9]. PROMIS is presently investigating CAT applications. It appears likely that the full potential of CAT applications requires calibration of additional items at the floor and at the ceiling to further improve the range of coverage. These items are presently being evaluated. Moreover, CAT requires electronic administration in real time, and the logistics are more cumbersome than with traditional pencil and paper administrations.

Theoretically, a nearly equivalent result may be obtained by using brief forms generated from the same calibrated item bank and tailored for strong reliability in a particular severity (theta) range matched to the study population. Such brief forms may also be developed through simulated CAT research, where the paths most frequently chosen identify the best items [8,9].

These data raise the issue of floor and ceiling effects [9]. PROMIS has defined the physical function domain as "Physical Function" rather than "Disability", expanding the conceptual basis to both increments in ability and decrements in ability. The predominant "gaps" in the present PROMIS PF item bank are in "ceiling" activities above the population average and at the "floor" represented by institutionalized populations. The more immediate problem is the ceiling, where more than 20% of these RA patients had zero HAQ scores. In healthy aging studies, over 50% may be at this ceiling. This results in a substantial loss of information and reduces study power [22]. Yet, we must be able to assess Physical Function above the mean to study normal or nearly normal subjects where detection of improvement is possible only if the scale permits above average scores.

Additional advantages accrue to PROMIS instruments that strengthen our confidence in recommending their use. The PROMIS process [12], beginning with item improvement, is directed at enabling instruments that are more patient-centered, validly translatable, have better clarity in diversely educated groups, have less Differential Item Functioning (DIF) across subgroups and are focused on supporting efficiency in clinical research. We, historically involved with the HAQ and PF-10, currently recommend the PROMIS PF 20 as the best available instrument for clinical studies with Physical Function endpoints.

To our knowledge, this is the first randomized study of alternative PRO instruments to document positive results with IRT-based instruments over traditional ones. We all are used to studies comparing treatments, not instruments, where effect sizes are often 0.6 or higher, rather than 0.06. This requires a different perspective, since improved PRO instruments are not interventions, but are more precise outcome measurement tools, much like a more precise sedimentation rate or more precise measurement of blood pressure. The improved precision effect may be smaller than the Minimally Clinically Important Differences (MCID) for interventions. They do, however, make it easier to detect whether an MCID is present or not. This, in turn, results in more efficient clinical research, requiring fewer subjects, fewer centers, shorter recruitment periods, reduced time to study completion, and easier monitoring of the trial, all very important considerations.

## Conclusions

The "present" of Physical Function assessment embodies new instruments derived from improved items and employing better item selection, which in turn results in lowering sample size requirements and improving efficiency. If 60 billion dollars is spent annually in the United States on clinical trial research and half of this (30 billion) is driven by per patient costs as opposed to administrative costs, and if one-fourth of these (7.5 billion) use quality-of-life patient-reported outcomes (for example, physical function, pain, fatigue, psychological distress) as primary endpoints, and if sample size requirements may be readily reduced by a factor of 2, then the unnecessary costs for using larger than necessary sample sizes may be in the range of three to eight billion dollars in the United States annually [23,24].

The "future" will include items and instruments that assess the floor and the ceiling and items and instruments tailored to specific ranges of functional ability. This will encourage broader functional status assessment and will enable CAT instruments with even broader coverage and greater precision of measurement.

## Abbreviations

CAT: computerized adaptive testing; HAQ: Health Assessment Questionnaire; HAQ-DI: Health Assessment Questionnaire Disability Index; IRT: item response theory; IRT-Based: items which have been calibrated by IRT within our item bank so that the better-performing items may be selected; Item-Improved: changes to items intended to improve their performance by altering parameters such as time frame and response options while retaining the same item stem; MCID: minimal clinically important difference; MDD: minimum detectable difference; NIH: National Institutes of Health; PF: Physical Function; PF-10: Physical Function 10-item short form from the SF-36; PROMIS: Patient-Reported Outcomes Measurement Information System; RA: rheumatoid arthritis.

## Competing interests

The authors declare that they have no competing interests.

## Authors' contributions

JF conceived and planned the study and participated in all aspects of its development. EK participated throughout, with particular emphasis upon analytic issues. MR participated throughout, and was responsible for IRT analyses and interpretation. BL participated throughout, and was responsible for many analyses and statistical issues. BB participated in design, analysis and manuscript development. All authors read and approved the final manuscript.
